# Endogenous glucocorticoids are required for normal macrophage activation and gastric *Helicobacter pylori* immunity

**DOI:** 10.1152/ajpgi.00114.2024

**Published:** 2024-07-23

**Authors:** Stuti Khadka, Sebastian A. Dziadowicz, Xiaojiang Xu, Lei Wang, Gangqing Hu, Javier A. Carrero, Richard J. DiPaolo, Jonathan T. Busada

**Affiliations:** ^1^Department of Microbiology, Immunology, and Cell Biology, West Virginia University School of Medicine, Morgantown, West Virginia, United States; ^2^Department of Pathology and Laboratory Medicine, Tulane University School of Medicine, New Orleans, Louisiana, United States; ^3^Department of Molecular Microbiology and Immunology, Saint Louis University School of Medicine, Saint Louis, Missouri, United States

**Keywords:** glucocorticoid, Helicobacter pylori, macrophage, pyloric metaplasia

## Abstract

Glucocorticoids are steroid hormones well known for their potent anti-inflammatory effects. However, their immunomodulatory properties are multifaceted. Increasing evidence suggests that glucocorticoid signaling promotes effective immunity and that disruption of glucocorticoid signaling impairs immune function. In this study, we conditionally deleted the glucocorticoid receptor (GR) in the myeloid lineage using the *LysM-Cre* driver (myGRKO). We examined the impact on macrophage activation and gastric immune responses to *Helicobacter pylori*, the best-known risk factor of gastric cancer. Our results indicate that, compared with wild type (WT), glucocorticoid receptor knockout (GRKO) macrophages exhibited higher expression of proinflammatory genes in steroid-free conditions. However, when challenged in vivo, GRKO macrophages exhibited aberrant chromatin landscapes and impaired proinflammatory gene expression profiles. Moreover, gastric colonization with *H. pylori* revealed impaired gastric immune responses and reduced T cell recruitment in myGRKO mice. As a result, myGRKO mice were protected from atrophic gastritis and pyloric metaplasia development. These results demonstrate a dual role for glucocorticoid signaling in preparing macrophages to respond to bacterial infection but limiting their pathogenic activation. In addition, our results support that macrophages are critical for gastric *H. pylori* immunity.

**NEW & NOTEWORTHY** Signaling by endogenous glucocorticoids primes macrophages toward more robust responses to pathogens. Disruption of glucocorticoid signaling caused dysregulation of the chromatin landscape, blunted proinflammatory gene activation upon bacterial challenge, and impaired the gastric inflammatory response to *Helicobacter pylori* infection.

## Graphical abstract

**Figure:**
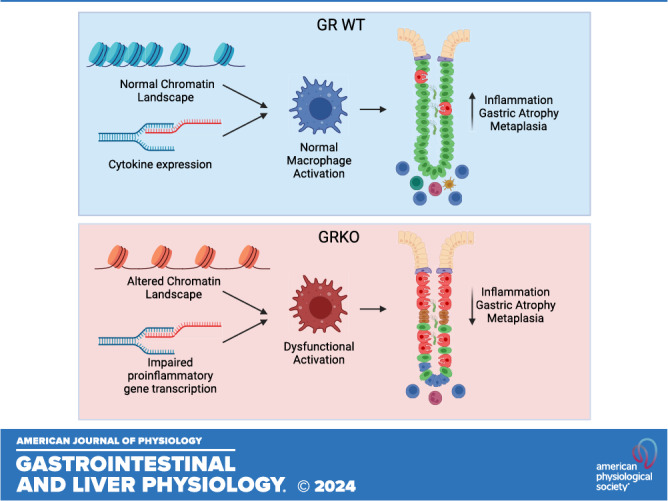

## INTRODUCTION

Glucocorticoids are steroid hormones produced as the end product of the hypothalamic-pituitary-adrenal axis. They signal through the glucocorticoid receptor (NR3C1, hereafter GR), a ligand-dependent transcription factor, regulating a wide array of cellular and physiological functions. Clinically, glucocorticoids are widely used to treat a host of inflammatory disorders, where they limit the activation and cytokine production of pathogenically activated immune cells (1). Their common clinical use has contributed to a dogmatic perception that glucocorticoids are purely anti-inflammatory. However, glucocorticoids exert diverse immunomodulatory effects, both inhibiting and enhancing aspects of immunity (2). Recent studies have found that signaling by glucocorticoids enhances T cell antigen specificity, regulates B cell homing, and enhances macrophage responses to lipopolysaccharide (LPS) (3–6). However, the role of glucocorticoids in supporting normal immune functions is poorly understood.

Gastric cancer is the fifth most common cancer worldwide (7). Chronic infection by the bacterium *Helicobacter pylori* is the highest known risk factor of gastric cancer. *H. pylori* infection is extremely prevalent, affecting ∼50% of the global population. The infection causes chronic inflammation within the gastric mucosa, initiating a well-established histopathological cascade of gastric atrophy and metaplasia, eventually resulting in gastric adenocarcinoma (8). Gastric inflammation is ubiquitous among *H. pylori*-infected individuals, but only a small portion of infected individuals will develop gastric cancer (9). Inflammation is important for controlling gastric *H. pylori* burden, but more intense immune responses also drive gastric atrophy and may accelerate gastric cancer initiation (10).

Macrophages are critical for multiple facets of the immune response. Early responses from tissue-resident macrophages detect pathogen-associated molecular patterns (PAMPs) and release chemokines to attract other immune cells. Phagocytosis and cytokine release reduce bacterial burden but may also induce off-target damage to host cells. Moreover, macrophage antigen presentation is important for generating T cell responses. Numerous studies have demonstrated that macrophages are essential during anti-*Helicobacter* immune responses. Macrophages control *H. pylori* gastric burden, and impaired macrophage function leads to higher *H. pylori* bacterial loads (11). However, macrophages also promote gastric atrophy, and macrophage depletion reduces *H. pylori*-associated gastric atrophy (12). Macrophage-derived cytokines also have been implicated in promoting pyloric metaplasia (PM) development (a putative preneoplastic lesion), and macrophage depletion prevents PM development following gastric injury (13–15). Thus, macrophages simultaneously are critical for protective immunity against *H. pylori* and damage the gastric mucosa, driving gastric atrophy PM development.

We recently reported that glucocorticoids are master regulators of gastric inflammation and that systemic disruption of glucocorticoid production by adrenalectomy caused spontaneous gastric inflammation, leading to gastric atrophy and PM development (15). Macrophages were key drivers of these gastric pathologies, as macrophage depletion prevented gastric atrophy and metaplasia. However, GR expression is ubiquitous in the stomach, and the cell-intrinsic effects of glucocorticoid signaling on macrophages and their impact on gastric immunity are unclear. In this study, we utilized the Cre-Lox system to specifically delete the GR from the myeloid lineage (myGRKO) and investigated the subsequent impact on macrophage activation and anti-*H. pylori* immunity. We found that glucocorticoid receptor knockout (GRKO) macrophages were dysfunctional, exhibiting aberrant chromatin accessibility and transcriptional responses following bacterial challenge. Moreover, myGRKO mice exhibited impaired gastric inflammation and reduced gastric atrophy following infection challenge with either *H. pylori* or *H. felis.* These results demonstrate a critical role for glucocorticoids in promoting normal proinflammatory macrophage function and that macrophages play a crucial role in coordinating gastric anti-*Helicobacter* immune responses.

## MATERIALS AND METHODS

### Animal Care and Treatment

Mice were housed and maintained in a temperature- and humidity-controlled vivarium with 12:12-h light-dark cycles and provided with standard chow and water ad libitum. The GR was deleted from the myeloid lineage by crossing *Nr3c1^flox/flox^* (Jax Stock No. 021021) and homozygous *LysM-Cre* (Jax Stock No. 004781) purchased from the Jackson Laboratories. *Nr3c1^flox/flox^*;*LysM^+/Cre^* mice were considered myeloid GRKO (myGRKO), whereas *Nr3c1^flox/flox^;LysM^+/+^* littermates were used as wild-type (WT) controls. Both sexes were used for these studies. Routine genotyping was performed by TransnetYX. For gastric colonization with *Helicobacter,* mice were inoculated with 500 µL of Brucella broth containing 10 (9) colony-forming units (CFU) of either *H. pylori* or *H. felis* two times 24 h apart. For peritoneal macrophage isolation, mice received a single intraperitoneal injection of 1 mL of sterile 3% Brewer thioglycollate media (Sigma-Aldrich). After 4 days, peritoneal lavage was plated, and nonadherent cells were removed by washing with prewarmed 1× phosphate-buffered saline (PBS).

### Bacterial Preparation

The *H. pylori* Premouse Sydney Strain 1 (PMSS1) strain (16) (a gift from Manuel Amieva, Stanford University) and the *H. felis* CS1 strain (ATCC 49179) were grown on tryptic soy agar plates (BD Biosciences) with 5% defibrinated sheep blood (Hemostat Labs) and 10 µg/mL vancomycin (Alfa Aesar) under microaerophilic conditions at 37°C for 2 days. Bacteria were harvested and transferred to Brucella broth containing 10% fetal bovine serum and 10 µg/mL vancomycin and grown overnight at 37°C under microaerophilic conditions with agitation. Bacteria were centrifuged and resuspended in fresh Brucella broth without antibiotics before spectrophotometry. Bacterial numbers were determined by a standard curve of bacterial counts and optical density. Gram staining was used to confirm culture purity.

### Tissue Preparation

Mice were euthanized by cervical dislocation without anesthesia. Stomach tissue was processed for flow cytometry, immunostaining, and RNA isolation as previously described (17). Briefly, stomachs were opened along the greater curvature and washed in phosphate-buffered saline (PBS) to remove the gastric contents. One half of the gastric corpus was used for flow cytometry. A 4-mm biopsy punch of the gastric corpus greater curvature was collected for RNA isolation and immediately snap-frozen in liquid nitrogen. From the other half of the stomach, a biopsy was collected to confirm *Helicobacter* colonization, and the remainder was fixed overnight in 4% paraformaldehyde at 4°C. After fixation, stomachs were washed with PBS and cut into strips. A strip was put in 30% sucrose and embedded in optimal cutting temperature compound for cryosectioning, and another strip was transferred in 70% ethanol for routine processing and paraffin embedding. To confirm *H. pylori* infection, biopsies from the gastric corpus and pylorus were homogenized and plated on tryptic soy agar plates with 5% defibrinated sheep blood containing amphotericin, bacitracin, nalidixic acid, and vancomycin. *H. pylori* growth was confirmed by gram staining. To confirm *H. felis*, DNA was isolated from a gastric biopsy and subjected to conventional PCR using primers targeting *H. felis FlaA.*

### Immunofluorescence Staining

Five-micrometer-thick cryosections from the gastric corpus greater curvatures were stained for key epithelial cells and metaplasia markers. The sections were incubated with antibodies for H^+^-K^+^-ATPase (MBL International), MIST1 (Cell Signaling Technologies), or CD44v9 (Cosmo Bio) overnight at 4°C. Primary antibody was omitted as a negative control. Sections were incubated in secondary antibodies for 1 h at room temperature. Fluorescent conjugated *Griffonia simplicifolia* lectin (GSII; ThermoFisher Scientific) was added with secondary antibodies. Sections were mounted with Vectastain mounting medium containing DAPI (Vector Laboratories). Images were obtained using a Zeiss 710 confocal laser-scanning microscope (Carl-Zeiss GmbH) and running Zen Black imaging software. Parietal cells and chief cells were quantitated as previously described (15) using confocal micrographs captured with a ×20 microscope objective. The total area quantified was 2,208 µm × 2,208 µm. Cells were counted with the ImageJ (National Institutes of Health, Bethesda, MD) count tool. Cells that stained positive with anti-H^+^-K^+^ antibodies were identified as parietal cells, whereas cells that stained positive with anti-MIST1 antibodies and were GSII negative were identified as mature chief cells. Counts were normalized to image area to determine the cell number per 100 µm^2^. Images that contained gastric glands cut longitudinally were selected for counting.

### Flow Cytometry

Single-cell suspensions were collected from one half of the gastric corpus. Stomachs were washed in 1× PBS, minced, and incubated in Hanks’ balanced salt solution containing 5% fetal bovine serum and 5 mM EDTA on a shaker incubator at 37°C. The tissue was then incubated with DNase (0.5 mg/mL; Worthington Biochemical) and collagenase type IV (1 mg/mL; Worthington Biochemical) and disassociated by pushing through a 100-µm cell strainer. Debris was removed using a 40% OptiPrep gradient (Serumwerk). Cell counts were collected with a Countess III (Invitrogen). Cells were stained for 20 min at 4°C with CD45 (clone 104), B220 (clone RA3-6B2), CD3e (clone 145-2C11), CD11b (clone M1/70), F480 (clone BM8), MHCII (clone M5/114.15.2), Ly6G (1A8) (all from Bioledgend) and SiglecF (clone E50-2440; eBiosciences). 7-Amino-actinomycin D (Invitrogen) was added to label dead cells. Samples were analyzed on a BD Fortessa (BD Bioscience). The data were analyzed with Cytobank (Beckman Coulter). Cell numbers were calculated by multiplying cell percentages (obtained by flow cytometry) by the total live cell counts.

### ATACseq

Mice were intraperitoneally injected with sterile thioglycollate medium as described above. After 4 days, mice received a single intraperitoneal injection of 10 million *H. pylori* in 500 µL of sterile saline. After 3 h, peritoneal lavage was collected, and the cells were stained with CD45 (clone 104), CD11b (clone M1/70), F480 (clone BM8), and CX3CR1 (SA011F11) for 20 min at 4°C. 7-Amino-actinomycin D (ThermoFisher) was used to label dead cells. Live CD45+CD11b+F480+CX3CR1+ macrophages were sorted on a fluorescence-activated cell sorting (FACS) Aria III (BD Bioscience). ATACseq was performed with 50,000 cells. Assay for Transposase-accessible Chromatin (ATAC) libraries were prepared with the Tagment DNA Enzyme and Buffer Kit (Illumina), following the Omni-ATAC protocol (18). The ATAC libraries were separated by electrophoresis on a 2% agarose gel. A gel segment corresponding to ∼200–600 bp was excised and the DNA purified with the Qiagen MinElute Gel Extraction Kit (Qiagen). Library quality was evaluated with Agilent Bioanalyzer 2100. Libraries were sequenced to 40 million reads on a Nextseq 2000 (Illumina) by the Marshall University Genomics and Bioinformatics Core facility. ATACseq data analysis was described previously (19). Briefly, ATACseq reads were aligned to the mouse reference genome (mm10) by Bowtie 2 (20); ATACseq read enriched regions were identified by macs 3 (21), differentially accessible regions (DARs) by EdgeR 3 (22), target genes of DARs by Genomics region enrichment of annotations (GREAT, version 4.0.4; Stanford University) (23), and motif analysis for DARs by HOMER (24). Pathway analysis for target genes of DARs were conducted by Metascape (25). A *P* value cutoff of 0.05 was used for pathway and motif analysis to assess the subtle changes between unstimulated WT and GRKO macrophages. A *q* value cutoff of 0.01 was used for the *H. pylori*-stimulated groups.

### RNAseq

WT and GRKO peritoneal macrophages were stimulated and isolated as described above in *ATACseq*. RNA was isolated with the Microelute RNA isolation kit (Omega Bio-Tek) with on-column DNase treatment (Omega Bio-Tek), following the manufacturer's protocol. Libraries were prepared by the West Virginia University Genomics Core using the NEBNext Low Input/Single Cell RNA-Seq kit (New England Biolabs). Libraries were sequenced to 30 million reads on a Nextseq 2000 (Illumina) by the Marshall University Genomics and Bioinformatics Core facility. RNAseq data analysis followed procedures as previously described (19). RNAseq reads were aligned by subread v2.0.1 (26). Read summarization was performed by RefSeq and transcript annotation by featureCounts (27) and differentially expressed genes identified by EdgeR3 (22). Gene Set Enrichment Analysis (GSEA 4.3.2) was used to identify pathway annotation of the genes preferentially expressed in a test group compared with the reference group. For each comparison, a “rnk” file was prepared with the expressed genes [reads per kilobase per million mapped reads (RPKM) ≥ 3] and their corresponding log fold change and run against the Kyoto Encyclopedia of Genes and Genomes (KEGG) and Reactome pathway collections predefined in MSigDB (28). The enrichment score on the GSEA was calculated using 1,000 permutations. The resulting GSEA pathways were further analyzed and visualized with Cytoscape (3.10.0). The EnrichmentMap plug-in on Cytoscape was used to visualize the GSEA KEGG [false discovery rate (FDR) ≤ 0.01] and Reactome (FDR ≤ 0.05) pathways for building the network graphs. Additionally, pathway analysis was performed with Metascape for differentially upregulated genes in a test group compared with the reference group (25) (min expr 3, FDR ≤ 0.05).

### Cytokine Analysis

WT and knockout (KO) mice were intraperitoneally injected with sterile thioglycollate medium as described above. After 4 days, mice received a single intraperitoneal injection of 10 million *H. pylori* in 500 µL of sterile saline. After 8 h, peritoneal lavage was collected in 1.5 mL of sterile PBS. Cytokines were measured in peritoneal lavage with a Meso Scale Discovery U-PLEX array (Meso Scale Diagnostics), following the manufacturer’s instructions.

### RNA and DNA Isolation and qPCR

RNA was extracted from a homogenized stomach biopsy in TRIzol (ThermoFisher Scientific) and precipitated from the aqueous phase with an equal volume of 70% ethanol. The mixture was transferred to an RNA isolation column (Omega Bio-Tek), and the remaining steps were followed according to the manufacturer’s recommendations. Quantitative real-time polymerase chain reaction (qRT-PCR) was performed with the iTaq Universal Probes master mix (Bio-Rad), and PCR was run on a QuantStudio 4 (ThermoFisher Scientific). The following Taqman primer probes were used (ThermoFisher Scientific): *Nr3c1* (Mm00433832_m1), *Tnf* (Mm00443258_m1)*, Il6* (Mm00446190_m1), *Ifng* Mm01168134_m1), *Aqp5* (Mm00437578_m1), and *Gkn3* (Mm01183934_m1)*. Ppib* (Mm00478295_m1) was utilized as a reference gene. DNA was isolated by incubating gastric biopsies in 0.05 M NaOH at 98°C. The pH was neutralized with 1 M Tris buffer, and the crude DNA was then cleaned with a Zymo Research Corporation DNA Clean & Concentrator kit (Zymo Research), following the manufacturer’s instructions. Relative bacterial density was measured by quantitative PCR using the *H. pylori* 23S specific primer probes (Forward: AACAAGTACCGTGAGGGAAAG; Reverse GCAGTCCATCACCCTGATAAA; Probe: 56-FAM/ AACCGCAGT/ZEN/ GAGCGGAGTGAAATA/3IABkFQ/) (Integrated DNA Technologies). Cycle thresholds were normalized to mouse DNA *Ppib* (Mm00291049_cn).

### Western Blot

Peritoneal macrophages were isolated as described above. Adherent cells were lysed in sodium dodecyl sulfate (SDS) sample lysis buffer (Bio-Rad). Proteins were separated by gel electrophoresis on a 10% Tris-glycine gel (Bio-Rad), transferred to nitrocellulose membrane, and stained with anti-GR antibodies (D8H2; Cell Signaling Technologies) and anti-β-actin antibodies (MAB1501; Millipore Sigma) overnight at 4°C. After stringency washes, the blots were probed with fluorescent secondary antibodies and were imaged with an iBright 1500 (ThermoFisher Scientific).

### Statistical Analysis

**Figure 1.**
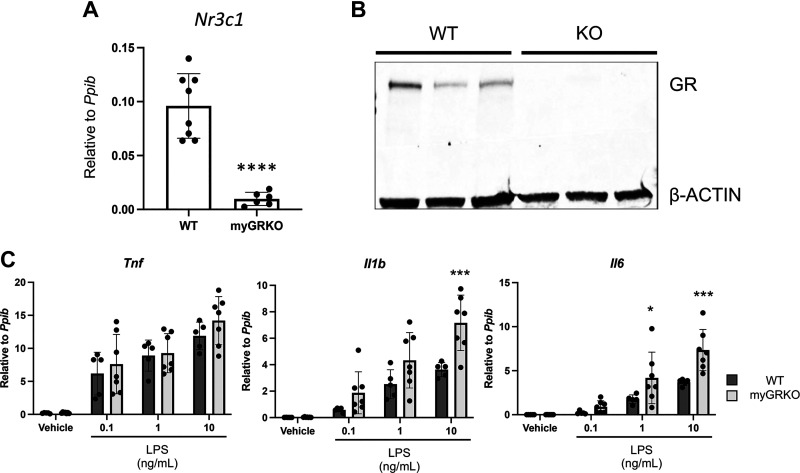
Loss of the glucocorticoid receptor (GR) poises macrophages to respond more aggressively to strong proinflammatory stimuli. *A* and *B*: quantitative RT-PCR (qRT-PCR) for *Nr3c1* (*A*) and Western blot (*B*) for the GR using RNA (*A*) or protein (*B*) isolated from adherent cells 24 h after isolation from thioglycollate-induced peritoneal macrophages from myGRKO (GR deleted in myeloid lineage) mice or wild-type (WT) littermate control mice. *C*: qRT-PCR of the indicated genes using RNA isolated from WT or GR knockout (GRKO) peritoneal macrophages stimulated with the indicated amount of LPS for 6 h. *n* ≥ 6 (*A*), *n* = 3 (*B* ), and *n* ≥ 5 (*C*). *P ≤ 0.05, ***P ≤ 0.001, ****P ≤ 0.0001.

GraphPad Prism 10 was used for the statistical analysis. An unpaired Student’s *t* test was used to compare two groups. One-way ANOVA with a post hoc Tukey *t* test was used when comparing more than two groups. Two-way ANOVA with Sidak’s multiple comparisons test was used when testing more than one variable (Fig. 1*C*). A *P* value of ≤0.05 was considered statically significant. All error bars are means ± standard deviation.

### Study Approvals

All mouse experiments were approved by the West Virginia University Animal Care and Use Committee.

## RESULTS

### Glucocorticoid Receptor Deletion Poises Macrophages to Respond More Aggressively to Lipopolysaccharide

To investigate the impact of glucocorticoid signaling on the macrophage response to proinflammatory stimuli, we deleted the GR from the myeloid lineage by crossing GR floxed mice (exon 3) with the *Lysm* promoter-driven Cre recombinase (3). To confirm the deletion of the GR, thioglycollate-induced peritoneal macrophages were isolated from myGRKO mice and WT littermate control mice. Quantitative RT-PCR revealed that *Gr* transcript levels were significantly reduced in KO macrophages compared with WT control mice (Fig. 1*A*). Similarly, Western blot revealed abundant GR protein in WT macrophages, but protein was not detectable in KO macrophages (Fig. 1*B*). Next, we examined how glucocorticoid signaling during monocyte-to-macrophage differentiation affected their subsequent activation. Thioglycollate-induced peritoneal macrophages were isolated from WT and myGRKO mice and cultured for 24 h in steroid-free conditions, followed by a 6-h stimulation with increasing concentrations of lipopolysaccharide (LPS). Relative transcript levels of the proinflammatory cytokines *Tnf*, *Il1b*, and *Il6* were measured by qRT-PCR. At a low LPS dose (0.1 ng/mL), there were no significant differences between the cytokine response of WT and GRKO macrophages (Fig. 1*C*). However, at moderate and high LPS doses, GRKO macrophages responded with significantly higher levels of *Il1b* and *Il6*, whereas *Tnf* levels were not significantly different at any LPS dose (Fig. 1*C*). These findings suggest that signaling by endogenous glucocorticoids during macrophage differentiation has lasting effects on macrophage sensitivity to strong proinflammatory stimuli and that GRKO macrophages are poised toward a hypercytokine response to strong proinflammatory stimuli.

### Unstimulated GRKO Macrophages Have Increased Chromatin Accessibility in Regions Adjacent to Proinflammatory Genes

**Figure 2.**
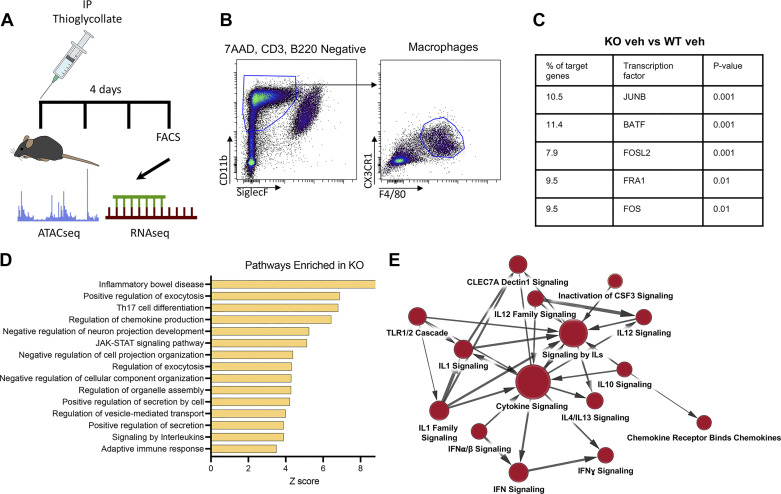
Glucocorticoid receptor knockout (GRKO) macrophages have increased chromatin accessibility in regions adjacent to proinflammatory genes. *A*: schematics showing experimental design. IP, intraperitoneal. *B*: gating strategy for isolating peritoneal macrophages. *C* and *D*: genomic regions that were significantly (*P* ≤ 0.05) more accessible in vehicle-treated GRKO macrophages were annotated by Genomics Region Enrichment of AnnoTations (GREAT). *C*: HOMER motif analysis identified transcription factor consensus sequences within these increased accessible regions. KO, knockout; veh, vehicle; WT, wild type. *D*: pathway analysis of the GREAT-annotated genes ranked by enrichment score. *E*: Cytoscape enrichment map summarizing the Gene Set Enrichment Analysis of RNAseq of vehicle-treated WT and GRKO macrophages. Red dots indicate enrichment in GRKO macrophages.

Our results demonstrate that GRKO macrophages have increased transcription of proinflammatory cytokines compared with WT macrophages cultured in steroid-free conditions. These results suggest that prior glucocorticoid signaling programs the macrophage response to proinflammatory stimuli. Glucocorticoid signaling through the GR is best known for its transcriptional activities, but recent evidence indicates that the GR signaling also shapes chromatin accessibility (29). Therefore, we tested whether the loss of glucocorticoid signaling altered chromatin accessibility. Thioglycollate-induced myGRKO and WT peritoneal macrophages were isolated by flow cytometry (Fig. 2, *A* and *B*). The chromatin landscape and transcriptome were assessed by ATACseq and RNAseq, respectively. Disruption of glucocorticoid signaling significantly affected the macrophage chromatin landscape from vehicle-treated mice, with 317 increased and 220 decreased differentially accessible regions (DARs) in vehicle-treated KO macrophages compared with vehicle-treated WT controls. HOMER motif analysis of the increased DARs revealed increased accessibility of AP1 family transcription factors such as JUNB and FOS (Fig. 2*C*). Genes that were spatially associated with DARs were annotated by the Genomic Regions Enrichment of Annotations Tool (GREAT) and assessed by Metascape pathway analysis. Pathway analysis predicted significant activation of inflammation-associated pathways such as inflammatory bowel disease, T cell activation, and chemokine production (Fig. 2*D*). Despite the dramatic alterations of chromatin accessibility adjacent to genes encoding proinflammatory mediators, RNAseq analysis revealed only subtle differences in the transcriptomes of vehicle-treated WT and GRKO macrophages with 187 differentially expressed genes (DEGs) when using a 0.05 *P* value cutoff and only 27 DEGs when using a 0.01 *P* value cutoff (Supplemental Fig. S1). Gene set enrichment analysis (GSEA) of the expressed genes demonstrated enrichment of several proinflammatory pathways in GRKO macrophages using the Reactome database. Cytoscape summary of the GSEA results demonstrated that “Cytokine signaling” was the most enriched node in GRKO macrophages (Fig. 2*E*). These data indicate that glucocorticoid signaling significantly impacts the macrophage chromatin landscape, with increased accessibility in regions adjacent to proinflammatory genes. Moreover, these results indicate that GRKO macrophages may be dysfunctional because of the persistent activation of proinflammatory gene networks.

### Glucocorticoid Signaling in Macrophages Is Required for Their Normal Activation to Bacterial Challenge

**Figure 3.**
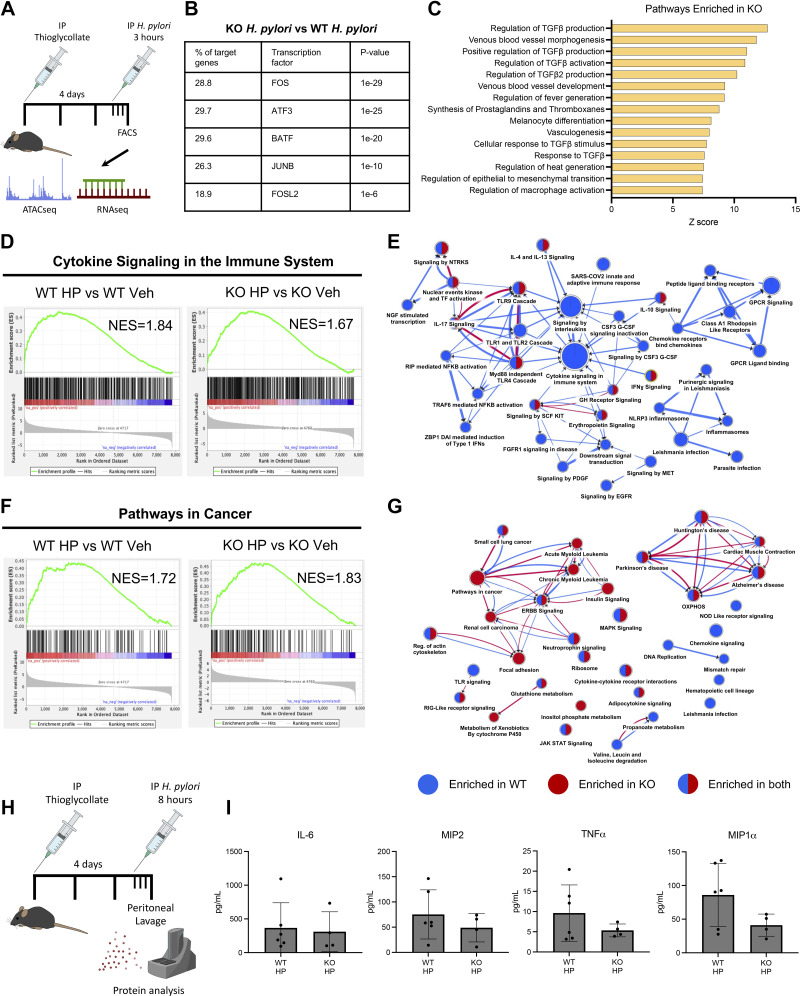
Glucocorticoid signaling in macrophages is required for their normal activation to inflammatory stimuli. *A*: schematics showing experimental design. IP, intraperitoneal. *B* and *C*: genomic regions that were significantly (*q* ≤ 0.01) more accessible in *Helicobacter pylori*-challenged glucocorticoid receptor knockout (GRKO) macrophages (KO) were annotated by Genomics Region Enrichment of AnnoTations (GREAT). *B*: HOMER motif analysis identified TF consensus sequences within these increased accessible regions. *C*: pathway analysis of the GREAT-annotated genes ranked by enrichment score. *D* and *F*: Gene Set Enrichment Analysis (GSEA) of the indicated Reactome (*D*) and Kyoto Encyclopedia of Genes and Genomes (KEGG; *F*) pathways comparing enrichment in activated wild-type (WT) and GRKO macrophages. NES, normalized enrichment score; Veh, vehicle. *E* and *G*: Cytoscape enrichment map summarizing the Reactome (*E*) and KEGG (*G*) GSEA results. Blue dots indicate enrichment in WT, and red dots indicate enrichment in GRKO macrophages. Mixed dots indicate enrichment in both groups. *H*: cytokine measurement from peritoneal lavage 8 h after challenge with *H. pylori. I*: protein concentration of the indicated cytokines. *n* ≥ 4.

Signaling by endogenous glucocorticoids is critical for normal macrophage function. Previous studies have found that GR deletion from the myeloid compartment leads to increased mortality to LPS sepsis challenge (30), whereas others have reported impaired cytokine production and wound healing in models of myocardial infarction (31). Our results indicate that in steroid-free conditions GRKO macrophages respond more aggressively to strong inflammatory stimuli. Next, we examined how these cells responded in vivo acute challenge by *H. pylori*. Endogenous steroid conditions are impossible to recapitulate in vitro. Therefore, we developed a novel in vivo challenge model, using thioglycollate to recruit monocyte-derived macrophages to the peritoneum followed by an intraperitoneal injection of live *H. pylori* at a 1:2 macrophage-to-bacteria ratio. The macrophages were collected by flow cytometry 3 h posttreatment (Fig. 3*A*). ATACseq revealed that *H. pylori* treatment induced massive chromatin reorganization, exacerbating the baseline differences between vehicle-treated macrophages with 3,204 increased and 1,260 decreased DARs in KO macrophages compared with WT *H. pylori*-treated macrophage controls. HOMER motif analysis of the increased DARs indicated a massive increase in binding sites for AP1-associated transcription factors (Fig. 3*B*). Pathway analysis of GREAT-annotated genes was used to describe the DARs between stimulated WT and KO macrophages. Within the top 15 pathways were annotations such as fever generation, prostaglandin synthesis, and regulation of macrophage activation (Fig. 3*C*). These enriched pathways suggested that the loss of glucocorticoid signaling exacerbates proinflammatory gene expression. Perplexingly, though, many of the upregulated networks included TGFβ signaling and regulation of macrophage activation, suggesting the parallel activation of anti-inflammatory gene networks to compensate for the loss of glucocorticoid regulation. Next, we compared how *H. pylori* challenge affects the macrophage transcriptomes. *H. pylori* challenge caused significant enrichment of inflammatory pathways in both WT and GRKO macrophages compared with their respective vehicle-treated controls. Surprisingly, GSEA revealed that inflammatory-associated pathways such as “cytokine signaling in the immune system” were more enriched in WT macrophages compared with KO [normalized enrichment score (NES) 1.84 vs. 1.67] (Fig. 3*D*). Cytoscape analysis of the enriched pathways from the Reactome database indicated that the activation of inflammatory gene sets was impaired in the *H. pylori-*challenged GRKO macrophages (Fig. 3*E*). Interestingly, GSEA using the Kyoto Encyclopedia of Genes and Genomes (KEGG) database indicated that several cancer-associated gene networks were significantly enriched in GRKO macrophages (Fig. 3, *F* and *G*). Because the RNAseq indicated that myGRKO macrophages exhibited defective activation of proinflammatory gene pathways, we next collected peritoneal lavage 8 h after mock or *H. pylori* challenge and measured cytokine levels (Fig. 3*H*). Some cytokines, such as IL-6 and MIP2, were equally induced in WT and KO mice (Fig. 3*I*). In contrast, MIP1α and TNFα were modestly elevated in WT mice compared with KO mice, although the differences did not rise to the level of statistical significance. Taken together, these data indicate that loss of glucocorticoid signaling in macrophages reshapes the chromatin landscape and impairs activation of proinflammatory genes.

### Loss of Glucocorticoid Signaling in the Myeloid Compartment Blunts the Gastric Inflammatory Response to *H. pylori* Infection

**Figure 4.**
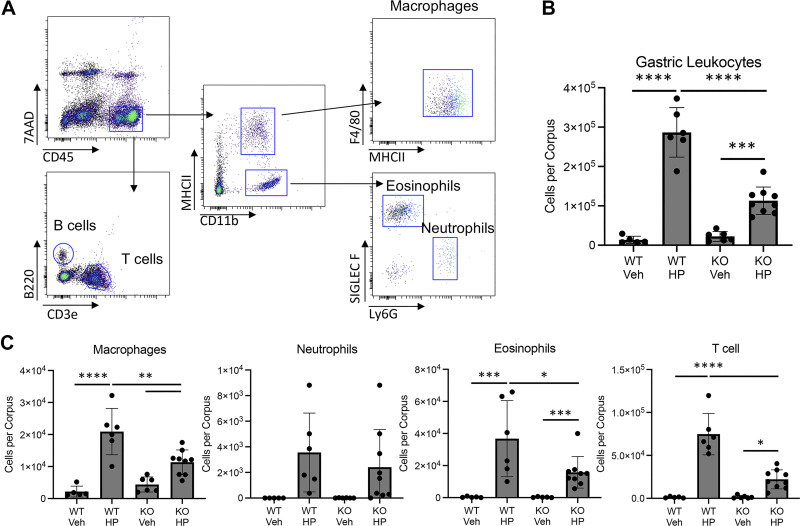
Loss of glucocorticoid signaling in the myeloid compartment impairs the gastric inflammatory response to *Helicobacter pylori* infection. *A*: representative flow cytometry plots demonstrating the gating strategy. *B* and *C*: flow cytometry analysis of the indicated cell types within the gastric corpus 2 mo after vehicle treatment (Veh) or *H. pylori* infection (HP). KO, knockout; WT, wild type. *n* ≥ 5. *P ≤ 0.05, **P ≤ 0.01, ***P ≤ 0.001, ****P ≤ 0.0001.

Our transcriptomics analysis indicated that the disruption of glucocorticoid signaling impaired macrophage activation in response to bacterial challenge. Macrophages are central to the gastric inflammatory response to *H. pylori* infection (12). Therefore, we next examined how myGRKO mice responded to chronic gastric *H. pylori* colonization. WT and myGRKO mice were infected with *H. pylori*, and inflammation within the gastric corpus was assessed 2 mo postcolonization. Flow cytometry was used to determine gastric immune infiltration (Fig. 4*A*). Tissue-resident leukocytes were similar in vehicle-treated WT and myGRKO stomachs (Fig. 4*B*). *H. pylori* colonization induced significant gastric leukocyte infiltration in both genotypes, but infiltration was significantly reduced in myGRKO stomachs (Fig. 4*B*). Within the myeloid compartment, macrophage and eosinophil recruitment, but not neutrophils, were significantly blunted in myGRKO mice compared with WT controls (Fig. 4*C*). Interestingly, T cell recruitment was also significantly impaired in myGRKO mice, suggesting that normal macrophage function is critical for coordinating the T cell response to *H. pylori* infection. Despite reduced inflammation in myGRKO mice, qPCR of *H. pylori* 23S revealed that bacterial density was not significantly different compared with WT mice (Supplemental Fig. S2). These data demonstrate that glucocorticoid signaling in the myeloid compartment is critical for mounting an immune response to *H. pylori* infection and provide increasing evidence that GRKO macrophages are dysfunctional.

### myGRKO Mice Are Protected from *H. pylori*-Induced Gastric Atrophy

**Figure 5.**
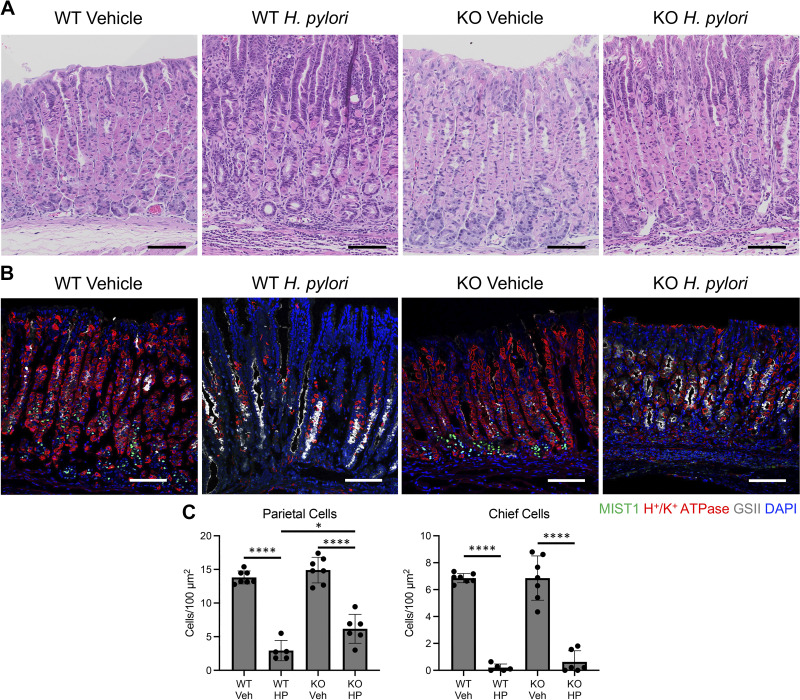
Disruption of glucocorticoid signaling in the myeloid compartment protects from *Helicobacter pylori*-induced gastric atrophy. *A*: representative hematoxylin-eosin (H&E) micrographs of the gastric corpus lesser curvature. KO, knockout; WT, wild type. Scale bars, 100 µm. *B*: representative immunostaining. Sections were probed with antibodies against the MIST1 (mature chief cells, green), the H^+^-K^+^-ATPase (parietal cells, red), and the *Griffonia simplicifolia* lectin (GSII; mucous neck cells, gray). Nuclei were stained with DAPI. Scale bars, 100 µm. *C*: quantitation of the number of parietal cells and chief cells (*n* ≥ 5 mice/group). HP, *H. pylori*; Veh, vehicle. Data are means ± SD. *P ≤ 0.05 and ****P ≤ 0.0001.

Chronic inflammation in response to *H. pylori* infection damages the gastric epithelium, driving metaplasia development and gastric cancer initiation. We found that the loss of glucocorticoid signaling in macrophages impairs their activation and blunts gastric T cell recruitment (Fig. 4). Next, we investigated how glucocorticoid signaling affected the development of *Helicobacter-*induced gastric atrophy and metaplasia. WT and GRKO mock-infected mice did not exhibit any noticeable differences in gross morphology within the gastric corpus (Fig. 5*A*). In WT mice, *H. pylori* colonization induced significant inflammation and gross atrophy of the gastric glands (Fig. 5*A*). However, the gastric atrophy was noticeably blunted in GRKO mice. Next, tissue sections from the gastric corpus lesser curvature were immunostained to label mucous neck cells, parietal cells, and chief cells. *H. pylori* infection induced significant parietal and chief cell atrophy in both WT and GRKO stomachs (Fig. 5, *B* and *C*). However, parietal cell atrophy was significantly blunted in GRKO mice compared with WT-infected control mice. The reduced gastric atrophy in GRKO mice was consistent with the impaired inflammation noted in Fig. 4.

### myGRKO Mice Are Protected from *Helicobacter felis*-Induced Chronic Atrophic Gastritis and Metaplasia

**Figure 6.**
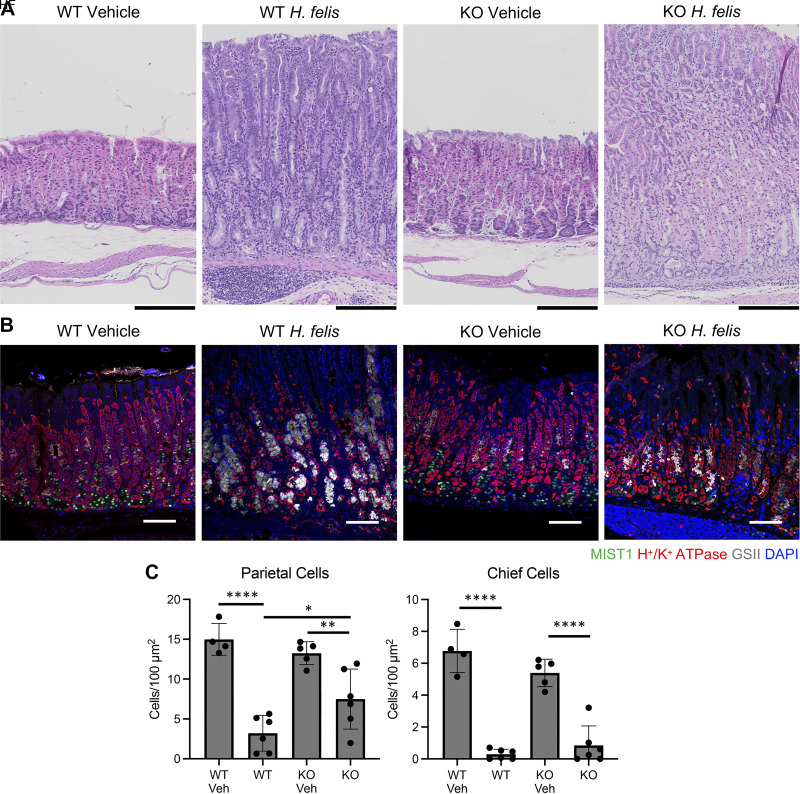
Disruption of glucocorticoid signaling in the myeloid compartment protects from *Helicobacter felis*-induced gastric atrophy. *A*: representative hematoxylin-eosin (H&E) micrographs of the gastric corpus lesser curvature. KO, knockout; WT, wild type. Scale bars, 100 µm. *B*: representative immunostaining. Sections were probed with antibodies against the MIST1 (mature chief cells, green), the H^+^-K^+^-ATPase (parietal cells, red), and the *Griffonia simplicifolia* lectin (GSII; mucous neck cells, gray). Nuclei were stained with DAPI. Scale bars, 100 µm. *C*: quantitation of the number of parietal cells and chief cells (*n* ≥ 5 mice/group). HF, *H. felis*; Veh, vehicle. Data are means ± SD. *P ≤ 0.05, **P ≤ 0.01, ****P ≤ 0.0001.

**Figure 7.**
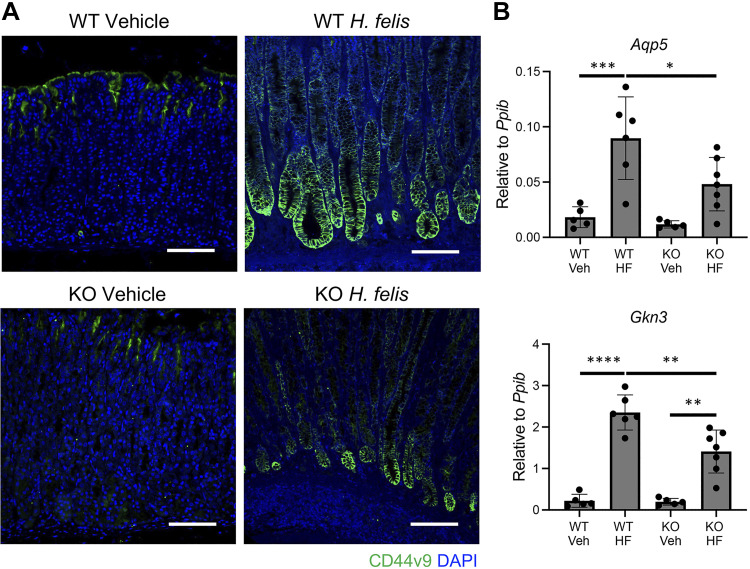
Deletion of the glucocorticoid receptor attenuates *Helicobacter felis*-induced pyloric metaplasia. Tissues and RNA were isolated from the gastric corpus greater curvature of wild-type (WT) and myGRKO (KO) mice 6 mo post-*H. felis* colonization. *A*: representative immunostaining for the pyloric metaplasia marker CD44v9. Nuclei are stained with DAPI. Scale bars, 100 µm. *B*: quantitative RT-PCR of the indicated pyloric metaplasia marker genes. HF, *H. felis*; Veh, vehicle. *n* ≥ 5. *P ≤ 0.05; **P ≤ 0.01, ***P ≤ 0.001, ****P ≤ 0.0001.

The *H. pylori* PMSS1 strain elicits mild inflammatory responses in mice (32, 33). Therefore, we next infected mice with *Helicobacter felis*, which induces severe gastric inflammation, atrophy, and metaplasia (33–35). Compared with mock-infected control mice, *H. felis* infection induced significant inflammation and gastric corpus remodeling in WT and myGRKO mice (Fig. 6*A*). However, gastric atrophy was more extensive in WT mice, with a significant reduction of parietal cell, the complete absence of chief cells, and a massive expansion of GSII+ mucous cells (Fig. 6, *B* and *C*). In contrast, gastric atrophy was notably more subdued in *H. felis-*infected myGRKO mice (Fig. 6). Parietal cell atrophy was significantly limited in myGRKO mice compared with WT-infected control mice (Fig. 6, *B* and *C*). Finally, we examined how the infection affected pyloric metaplasia development. In WT mice, *H. felis* infection caused widespread expression of CD44v9, extending to the base of gastric corpus glands (Fig. 7*A*). Moreover, there was a significant increase in the PM transcripts *Aqp5* and *Gkn3* compared with mock-infected controls (Fig. 7*B*). In contrast, CD44v9 expression was notably reduced in myGRKO stomachs, and *Aqp5* and *Gkn3* expression was significantly blunted compared with WT controls (Fig. 7). These results demonstrate that macrophage dysfunction impairs the gastric inflammatory response to *Helicobacter* infection and indicates that glucocorticoid signaling in macrophages is required for their normal responsiveness to bacterial infection.

## DISCUSSION

Our data demonstrate that glucocorticoid signaling is required for the normal macrophage response to *H. pylori*. Loss of glucocorticoid signaling caused reorganization of the chromatin landscape, increasing accessibility in regions adjacent to proinflammatory genes, in both resting and stimulated macrophages. Upon challenge with *H. pylori,* GRKO macrophage activation was impaired, and their transcriptional response was skewed toward cancer-associated gene sets. Within the stomach, myGRKO mice exhibited defective immunity against *H. pylori* infection, with fewer inflammatory cells and reduced atrophy of corpus glands. Finally, when myGRKO mice were chronically infected with *H. felis*, they exhibited lower levels of gastric atrophy and pyloric metaplasia. Together, these results suggest that glucocorticoid signaling poises macrophages for an effective response to pathogens and demonstrate that glucocorticoid signaling is required for normal macrophage activation.

Glucocorticoids have been clinically used for over half a century and remain one of the most widely used drugs for combating inflammatory diseases (36). There is a formidable body of literature on the anti-inflammatory effects of glucocorticoid treatments. Their wide-ranging immunomodulatory effects are achieved through cell type-specific transcriptional changes (37). The effects of glucocorticoid treatment are primarily anti-inflammatory, where they oppose proinflammatory cytokine transcription, inhibit B cell receptor and T cell receptor signaling, suppress pattern recognition receptor expression and activation, oppose antigen presentation, etc. (1). However, many of the immunomodulatory effects of glucocorticoids are dictated by ligand concentration, and endogenous glucocorticoids play a more nuanced role in regulating immune activation. In macrophages, high doses of glucocorticoids strongly suppress the transcription of proinflammatory genes, whereas low doses promote the transcription of proinflammatory genes (4). Similarly, low doses of glucocorticoids delivered before an inflammatory challenge enhanced the macrophage response to LPS (38), suggesting that glucocorticoids are important for preparing the immune system for noxious stimuli.

In this study, we utilized a novel macrophage challenge paradigm by exposing macrophages to inflammatory stimuli in vivo. Thioglycollate-induced macrophages are primarily monocyte derived (39), and WT cells differentiated in vivo under the influence of endogenous glucocorticoid, whereas GRKO macrophages were insensitive to the direct effects of endogenous corticosteroids. In a previous study, Bhattacharyya et al. (30) reported that myGRKO mice died from septic shock when challenged with LPS, whereas WT control mice survived the challenge. Thus, our initial expectation was that GRKO macrophages would develop a hyperactive response to an intraperitoneal *H. pylori* challenge. However, GRKO macrophages exhibited defective activation of proinflammatory genes and instead exhibited enrichment of several cancer-associated gene networks. Moreover, myGRKO mice mounted a weaker gastric inflammatory response to *H. pylori* colonization. These results demonstrate a dichotomy in the immunomodulatory effects of endogenous glucocorticoids. Bhattacharyya et al. (30) reported that endogenous glucocorticoids are critical for limiting inflammatory intensity to a high dose of LPS and preventing septic shock. In contrast, our results indicate that, during steady-state conditions, endogenous glucocorticoids function to prime the macrophage response and are critical for effective immunity against *H. pylori*. The differences in the type and intensity of the inflammatory stimuli may partially explain these context-dependent findings. LPS challenge is a strong proinflammatory stimulus, and glucocorticoids likely function to limit immune activation and cytokine production. In contrast, *H. pylori* is a chronic bacterial infection that induces limited systemic inflammation (40). In this environment, our results suggest that glucocorticoids support a more effective antibacterial immune response. Glucocorticoid signaling impacts a wide array of cellular responses, and it is estimated that glucocorticoids regulate up to 20% of expressed genes (41). However, the cell type-specific transcriptional changes regulated by glucocorticoids are primarily determined through preexisting chromatin accessibility patterns (42). Ligand-activated GR recruits several coactivators and corepressors that affect the chromatin landscape, including histone methyltransferases and acetyltransferases as well as the SWI/SNF chromatin remodeling complex (29, 43). We found that *H. pylori* stimulation induced dramatic differences in the chromatin landscape in WT versus GRKO macrophages. Interestingly, many of the increased DARs in KO macrophages contained AP1 binding motifs, a transcription factor complex that drives the expression of many proinflammatory cytokines. The AP1 complex is a well-known target of GR transrepression, and the increased accessibility of AP1 motifs is likely a direct effect of the loss of the GR (44). Interestingly, pathway analysis of the increased DARs in GRKO macrophages indicated a striking activation of TGFβ signaling. Similar findings were reported by Galuppo et al. (31), who found that *Tgfb1* expression was increased in GRKO macrophages. TGFβ is an anti-inflammatory cytokine that suppresses macrophage activation and promotes alternative macrophage polarization (45). The increase in TGFβ signaling may represent a compensatory mechanism to oppose the hyperactivation caused by the loss of glucocorticoid signaling.

Within the stomach, macrophages act as a double-edged sword. During steady-state conditions, they are the most abundant tissue-resident leukocytes in the gastric corpus, where they maintain tissue homeostasis (17). However, monocyte-derived macrophages produce damaging cytokines following gastric injury that exacerbate tissue and promote pyloric metaplasia development (14, 15). During *H. pylori* infection, macrophages simultaneously control bacterial loads and contribute to gastric atrophy. Activation of proinflammatory M1-like macrophages is associated with reduced bacterial colonization but also increased epithelial damage and gastric atrophy (46, 47). In contrast, factors that impair macrophage activation led to increased bacterial loads and reduced gastric atrophy (11, 48). Our results demonstrate that loss of glucocorticoid signaling impaired the macrophage response to *H. pylori* and *H. felis.* This impaired activation reduced inflammatory damage to the gastric corpus, limiting gastric atrophy and metaplasia development.

The direct effects of macrophages and macrophage-derived cytokines on gastric atrophy are unclear. We have previously shown that systemic glucocorticoid depletion induces spontaneous gastric inflammation and PM and that macrophage depletion, through either clodronate liposomes or blocking monocyte infiltration in *Cx3cr1* KO mice, prevents these gastric pathologies (15). Similarly, work by Petersen et al. (13, 14) showed that macrophage-derived cytokines drive PM development following L635-induced gastric injury. Although these studies demonstrate that macrophages contribute to gastric atrophy progression, others have shown that *Helicobacter*-induced gastric atrophy is blocked in T cell-deficient mice (49). Thus, macrophage effects on *Helicobacter-*induced gastric atrophy may occur through coordinating the T cell responses. Supporting this notion, macrophage depletion in *H. pylori-*infected gerbils reduced lymphocyte infiltration and tertiary lymphoid tissue development (50). Here, we found that myGRKO mice had reduced gastric T cells. Thus, in addition to the cell-intrinsic macrophage dysfunction caused by defective glucocorticoid signaling, these effects also impair adaptive immunity.

In summary, glucocorticoid signaling is critical for multiple phases of the immune response, preparing the immune system to combat pathogens effectively while limiting the immune response of pathogenically activated immune cells. Our results show that endogenous glucocorticoid signaling impacts the macrophage transcriptome and chromatin landscape and that the loss of glucocorticoid signaling induces macrophage dysfunction. Although this study focused on cells with either intact or disrupted glucocorticoid signaling, the temporal effects of glucocorticoid signaling have important clinical relevance and were not addressed here. In addition, our findings underscore the critical role that gastric macrophages play in the immune response to *H. pylori,* where they impact the T cell response and promote gastric atrophy and metaplasia development. These findings, combined with previous reports (15, 17, 51), demonstrate that glucocorticoid signaling is important for regulating gastric immunity and may impact gastric cancer risk.

## DATA AVAILABILITY

Data will be made available upon reasonable request. The bulk RNAseq data and ATACseq data were deposited in the Gene Expression Omnibus (Accession numbers GSE253584 and GSE253583, respectively).

## SUPPLEMENTAL MATERIAL

10.6084/m9.figshare.26008294 Supplemental Figs. S1 and S2: https://doi.org/10.6084/m9.figshare.26008294.

## GRANTS

This work was supported by West Virginia University start-up funds (to J.T.B), the West Virginia University Cancer Institute, and from the National Institutes of Health (NIH) Grant P20GM121322 (to J.T.B.). The West Virginia University Microscope Imaging Facility, Flow Cytometry and Single Cell Core, and Genomics Core Facility receive support from NIH Grants P30GM103503, S10 OD028605, and U54 GM104942, respectively. The West Virginia University Bioinformatics Core Facility is supported by NIH Grants U54GM104942 and P20GM103434.

## DISCLOSURES

No conflicts of interest, financial or otherwise, are declared by the authors.

## AUTHOR CONTRIBUTIONS

S.K. and J.T.B. conceived and designed research; S.K., S.A.D., J.A.C., and J.T.B. performed experiments; S.K., S.A.D., X.X., L.W., G.H., J.A.C., R.J.D., and J.T.B. analyzed data; S.K., S.A.D., G.H., R.J.D., and J.T.B. interpreted results of experiments; S.K., X.X., L.W., G.H., and J.T.B. prepared figures; S.K. and J.T.B. drafted manuscript; S.K. and J.T.B. edited and revised manuscript; S.K., S.A.D., X.X., L.W., G.H., J.A.C., R.J.D., and J.T.B. approved final version of manuscript.
